# Sustained release glaucoma therapies: Novel modalities for overcoming key treatment barriers associated with topical medications

**DOI:** 10.1080/07853890.2021.1955146

**Published:** 2022-01-25

**Authors:** Aditya Belamkar, Alon Harris, Ryan Zukerman, Brent Siesky, Francesco Oddone, Alice Verticchio Vercellin, Thomas A. Ciulla

**Affiliations:** a Indiana University School of Medicine, Indianapolis, IN, USA; b Department of Opthalmology, Icahn School of Medicine at Mount Sinai, New York, NY, USA; c Department of Opthalmology, University of Miami Miller School of Medicine, Miami, FL, USA; d Department of Opthalmology, Fondazione Bietti, Rome, Italy; e Vitreoretinal Medicine and Surgery, Midwest Eye Institute, Indianapolis, IN, USA

**Keywords:** Glaucoma, glaucoma treatment, topical therapy, sustained release, adherence, nanotechnology

## Abstract

Glaucoma is a progressive optic neuropathy and a leading cause of irreversible blindness. The disease has conventionally been characterized by an elevated intraocular pressure (IOP); however, recent research has built the consensus that glaucoma is not only dependent on IOP but rather represents a multifactorial optic neuropathy. Although many risk factors have been identified ranging from demographics to co-morbidities to ocular structural predispositions, IOP is currently the only modifiable risk factor, most often treated by topical IOP-lowering medications. However, topical hypotensive regimens are prone to non-adherence and are largely inefficient, leading to disease progression in spite of treatment. As a result, several companies are developing sustained release (SR) drug delivery systems as alternatives to topical delivery to potentially overcome these barriers. Currently, Bimatoprost SR (Durysta^TM^) from Allergan plc is the only FDA-approved SR therapy for POAG. Other SR therapies under investigation include: bimatoprost ocular ring (Allergan) (ClinicalTrials.gov identifier: NCT01915940), iDose^®^ (Glaukos Corporation) (NCT03519386), ENV515 (Envisia Therapeutics) (NCT02371746), OTX-TP (Ocular Therapeutix) (NCT02914509), OTX-TIC (Ocular Therapeutix) (NCT04060144), and latanoprost free acid SR (PolyActiva) (NCT04060758). Additionally, a wide variety of technologies for SR therapeutics are under investigation including ocular surface drug delivery systems such as contact lenses and nanotechnology. While challenges remain for SR drug delivery technology in POAG management, this technology may shift treatment paradigms and dramatically improve outcomes.

## Graphical Abstract

**Figure:**
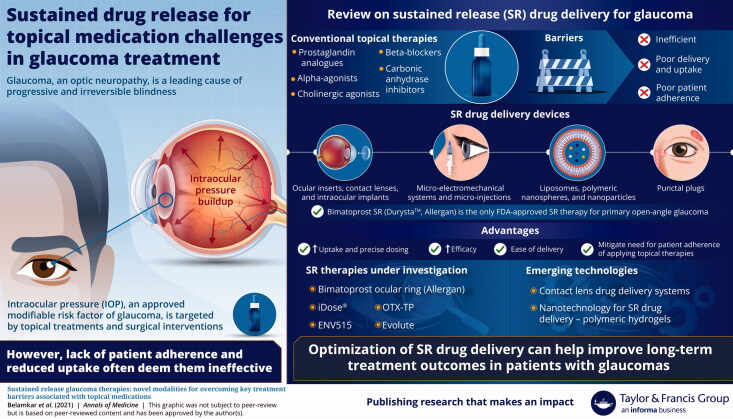

## Introduction

Glaucoma describes a family of progressive, degenerative optic neuropathies, characterized by the loss of retinal ganglion cells and ensuing structural alterations to the optic nerve head (ONH) and retinal nerve fibre layer [1]. These changes are typically associated with asymptomatic vision loss beginning in the midperiphery regions and moving centrally [1]. Glaucoma is a leading cause of irreversible blindness affecting over 76.0 million in 2020 with a predicted increase to 111.8 million in 2040; glaucoma has a prevalence of 3.54% in those aged 40–80, with the most common form of the disease, primary open-angle glaucoma (POAG), estimated at 3.05% [1,2]. The disease is characterized by an elevation in intraocular pressure (IOP), however, the past two decades of clinical findings have caused this definition to evolve from an IOP-only disease to a multifactorial optic neuropathy [3]. Still, IOP remains the only modifiable risk factor and is known to be effective in decreasing the cumulative probability of developing POAG and risk of progression in individuals with ocular hypertension (OHT) [1,4,5]. However, studies have estimated that IOP was lower than 22 mmHg in a quarter to one half of all individuals with glaucoma, necessitating the identification of other risk factors for diagnostic and treatment purposes [1].

### Risk factors

Although elevated IOP is an important risk factor for glaucoma, other identified risk factors include advanced age, high myopia, a positive family history of glaucoma, pseudoexfoliation, African descent, male gender, hypertension, diabetes mellitus, ocular perfusion pressure, and a cup-to-disk ratio greater than 0.7 [2,5–12]. The interactions between the factors remain enigmatic and a major focus of current research. Other factors associated with glaucoma include abnormalities in ocular blood flow and regulation, autoimmunity, retinal oxygen saturation, decreased intracranial pressure, and subsequently elevated translaminar cribrosa pressure difference [13–23]. The vascular hypothesis of glaucoma is an important idea connecting the alterations in hemodynamic biomarkers with other risk factors including race, gender, and diabetic status to explain disease progression, though it is not currently clear whether or not these vascular biomarkers are the primary insult or act secondary to the ongoing glaucomatous process [2,3,12,24–29]. While the disease manifestations are very similar, there may exist many, individualized pathological mechanisms for disease development and progression.

### Current glaucoma topical therapeutics

The current glaucoma treatment strategy is focused on lowering IOP with various classes and combinations of topical hypotensive medications and surgical interventions. Treatment is typically initiated in a stepwise fashion starting with single topical drug therapy, followed by multidrug combinations, and, if necessary, laser treatments and surgical intervention [30]. Topical medications can involve any of three mechanisms of action, including increasing aqueous outflow *via* the trabecular meshwork, increasing outflow *via* the uveoscleral pathway, or decreasing the production of aqueous humour. Topical therapies are divided into five major classes: prostaglandin analogues, beta-blockers, diuretics (carbonic anhydrase inhibitors), cholinergic agonists, and alpha-agonists [31]. Topical prostaglandin analogues and selective or non-selective beta-blockers are conventionally used as the first-line of treatment to reduce IOP, followed by alpha-agonists and topical carbonic anhydrase inhibitors [30–32]. Third- and fourth-line treatment options include cholinergic agonists and systemic, orally administered agents [30,33]. If initial monotherapies are not effective in controlling IOP and structural and visual parameters, other drugs with different mechanisms of action can be added to or replace initial therapies; combination therapies are also an effective option in patients who may struggle with adherence [30,31].

Beta-blockers were once held as the gold standard of topical hypotensive medications but have recently been replaced in this role by prostaglandin analogues. Beta-blockers reduce IOP by decreasing aqueous humour formation [30–32]. The adverse ocular side effects of beta-blockers are relatively few, limited to ocular irritation and allergic conjunctivitis, however, their systemic side effects pose major issues and include exacerbation or precipitation of asthmatic attack, respiratory failure, cardiac failure, bradycardia, hypotension, depression, and impotence [30,32,34]. Beta-blockers are advantageous in that they can be used once or twice-daily and do not affect pupil size or lens accommodation [32].

Prostaglandin analogues are highly effective hypotensives that function by reducing aqueous humour outflow resistance and thus increasing outflow through the uveoscleral pathway [30–32]. They have also been suggested to have a minor effect on classical outflow facility. They possess relatively few adverse systemic side effects, can be used only once daily, and do not affect pupil size or lens accommodation [32]. Prostaglandin analogues have been shown to cause iris pigmentation changes, hypertrichosis of eyelashes, and ocular inflammation; however, they are currently the primary choice for first line therapies as they have been shown to be superior to beta-blockers in reducing IOP and increasing persistency without the systemic side effects [30–41]. It is also theorized that these drugs may be more effective than beta-blockers in preventing acute pressure spikes due to their mechanism of increasing outflow rather than limiting aqueous humour production [30].

Second-line treatments for IOP reduction include alpha-agonists and topical carbonic anhydrase inhibitors. These classes of drugs are typically administered twice or thrice daily and are associated with few systemic and ocular side effects [30–32]. They are typically added to first-line therapies to further lower IOP in a multidrug approach or replace them for long-term sustained therapy [32]. Alpha-agonists function as hypotensives by increasing uveoscleral outflow and decrease aqueous flow; carbonic anhydrase inhibitors function through enzymatic inhibition in the aqueous humour production pathway [30,33]. These second-line treatments are commonly utilized in combination drugs which are thought to improve patient compliance and adherence [30,42].

Cholinergic agonists, also known as parasympathomimetic or miotic agents, are typically the last line of topical medications. These drugs are relatively inexpensive and associated with few systemic side effects, but they have also been associated with blurred vision, miosis, accommodative spasm, and retinal tears or detachments and must be used three or four time daily [32].

Rho-kinase inhibitors are a relatively newer class of drugs for glaucoma management that function to reduce IOP by directly affecting the trabecular meshwork and Schlemm’s canal which reduces the resistance to aqueous humour outflow. While these drugs may be a promising option for the clinical management of IOP, their novelty as topical therapies posit them outside the scope of this review.

While topical hypotensive medications are currently the best treatment option for lowering the risk of progression, there are still many issues with reliance on these including diurnal variations in IOP, alterations in ocular blood flow, and neuroprotection. Additionally, issues with patient adherence and inefficiencies in drug delivery *via* topical mechanisms further complicate this treatment modality.

### Issues with patient adherence

A key obstacle to glaucoma treatment with topical hypotensives is the lack of patient adherence to medication regimens. Studies have estimated 5% to 80% of patients deviate from their prescribed medication regimen, with most studies estimating approximately 70% to 80% are adherent through both self-reported and electronic monitoring methods [43–50]. Lack of adherence to medication regimens is an incredibly important clinical issue as these drugs represent the best option for limiting the risk of progression to blindness. Studies have correlated higher adherence rates with stable visual field test measurements and lack of progression [51,52].

The Glaucoma Adherence and Persistency study, a large-scale study conducted on adherence to glaucoma medications, found a mean medication possessional ratio (MPR) of 0.64 for 13,956 subjects [44]. This ratio is defined as the days of prescription supply dispensed divided by the number of days between first and last prescription refill and is known to be a robust measure of adherence over time that accurately reflects the proportion of patients who stop and restart medications [44]. Specifically, the study found that MPR was 0.56 for subjects who used a hypotensive medication unilaterally but was 0.70 for those who used a medication bilaterally [44]. Similarly, other studies have found nearly half of subjects discontinue topical therapies within six months, with just 55.6% of subjects taking greater than 75% of expected doses and only 37% refilling the initial medication three years after it was first dispensed [41,49]. Additionally, studies have found greater rates of adherence to simpler drug regimens and once-daily drugs [41,53].

Many factors and barriers have been identified to better understand the lack of adherence to glaucoma medication regiments including: forgetfulness, lack of self-efficacy (the ability of patients to be motivated), difficulty instilling eye drops, scepticism about glaucomatous vision loss and prevention of it, insufficient knowledge about glaucoma, complexity of the medication regimen, number of ophthalmologist visits in the first year, and demographic and sociographic variables [41,43,45–47,53,54]. A survey study by Newman-Casey et al. [45] found for each additional barrier cited as important, there was a 10% increased odds of being non-adherent. Additionally, the barriers with the highest odds ratio for non-adherence, adjusted for age, were forgetfulness, decreased self-efficacy, difficulty instilling eye drops, and difficulties with the medication schedule [45]. The study found subjects who were non-adherent were significantly younger than subjects who were adherent [45]. Although commonly expected to be a barrier for adherence, older age has not been consistently shown to be a risk factor for poor or non-adherence to glaucoma medication regiments, while comparisons of adherence rates based on race have yielded mixed results [52,54]. Improving adherence to glaucoma medication regimens remains a significant issue for clinicians in providing the best possible treatments for their patients.

### Inefficiencies of topical medications

Topical hypotensive therapies are also known to be inefficient, compounding the issues associated with patient adherence. On average, 20% of an individual dosage was shown to be wasted due to inefficient drug delivery by using more than one drop per dosing [55]. Some patients were found to require up to 3.7 drops per scheduled dose per eye [47,55]. Studies have also found the volume that can be administered to the ocular surface for absorption is limited to approximately 30 µL, resulting in a large proportion of the drug being wasted [56,57]. Furthermore, not only are topical doses cleared from the ocular surface within minutes, but they are also inefficiently absorbed across the corneal surface due to the small surface area and relative impermeability of the hydrophilic and lipophilic structures, resulting in approximately 1% to 10% bioavailability [56,58–62]. Drug uptake into the conjunctiva further reduces bioavailability [56,63]. Even if a patient has high rates of adherence, these inefficiencies in topical ocular drug delivery may result in futile control of IOP and subsequent disease progression. In order to overcome these challenges associated with patient adherence and topical application, a variety of interventions are currently being explored. Chief among these is the development of sustained release (SR) drug delivery systems, which represent an important avenue for the future of glaucoma and OHT disease management.

## Currently approved sustained release therapies

There is currently significant research being conducted on novel therapeutic drug delivery systems for the management of glaucoma due to aforementioned issues with topical medications, however, their success has largely been limited. Types of drug delivery systems being explored include ocular inserts, contact lenses, intraocular implants, microelectromechanical systems, liposomes, polymeric nanoparticles, nanospheres or microspheres, injectable systems, punctal plugs, pentablock copolymer gels, and microneedles, with injectable systems and punctal plugs being a major focus for the development of SR therapies [64]. To date, SR therapies have yet to be implemented clinically.

In March 2020, the field of glaucoma therapeutics hit a major milestone with the approval of a SR IOP-lowering ocular implant by the United States Food and Drug Administration (FDA) [65,66]. Bimatoprost SR, known commercially as Durysta^TM^, was introduced by Allergan plc (Dublin, Ireland) to allow for long-term, consistent treatment of glaucoma and to overcome adherence issues in many patients [65]. The implant consists of a 10-µg dose of bimatoprost, released in a non-pulsatile, steady-state fashion, held within a rod-shaped poly-D,L-sustained lactide-co-glycolide (PLGA) polymer matrix drug delivery system based on Allergan’s NOVADUR^®^ platform [65–69]. The implant can be inserted in the clinic or operating room and does not need to be removed as the PLGA polymer matrix is biodegradable [65,67,69]. Bimatoprost SR is placed intraocularly in the iridocorneal angle with a target duration of three to four months, however, studies did find that the implant could be efficacious for much longer [64,65,67–69]. Currently, the FDA has only approved the implant for single administration in patients. The implant also displayed a very favourable safety profile and positive treatment experience for patients [64,67–69]. Adverse events were found to be more common in eyes with implants and included conjunctival hyperaemia, foreign body sensation, and eye pain but were resolved after several days and associated with the implantation procedure rather than the implant [65,67–69]. Conjunctival hyperaemia with onset later than two days after the implantation procedure was associated with topical bimatoprost [68,69]. Central endothelial cell density loss, an important safety consideration, was only slightly greater in implant eyes than control eyes throughout each phase of the clinical trials [65,67–69].

Preclinical studies conducted in beagle dogs allow for an elucidation of pharmacokinetics and dynamics of the drug delivery system as well as its impact on the eye. Normotensive beagle dogs were randomized to receive Bimatoprost SR dosed with 30 µg or a sham injection. These dogs were found to display a reduced IOP that was maintained 66 days following baseline treatment [70]. Bimatoprost SR was also associated with a transient increase in episcleral venous pressure followed by a sustained decrease and dilation of aqueous outflow vessels [70]. This is a particularly relevant finding as the Goldmann equation suggests episcleral venous pressure to be an important contributor to IOP, with a reduction in this pressure directly associated with a reduction in IOP [70]. Bimatoprost is known to increase aqueous humour outflow through the uveoscleral pathway, and thus this finding may illustrate another avenue for the IOP-lowering effect of this drug delivery system [30–32]. Another study in normotensive dogs found that the dose-response curves for topical bimatoprost and Bimatoprost SR differed [71]. Topical bimatoprost demonstrated a U-shaped curve, with an increase in dose concentration to 0.1% resulting in reduced IOP-lowering efficacy, but, the curve for the Bimatoprost SR implant showed consistently greater IOP-lowering capacity as the dose strength was increased [71]. One potential explanation for this difference was that Bimatoprost SR administered the dose directly at the iris-ciliary body [72]. Bimatoprost concentration in the bulbar conjunctiva, eyelid margin, and periorbital fat were significantly reduced or undetectable in eyes with the implant compared to eyes administered with topical bimatoprost [72]. Additionally, peak drug concentrations in the dogs’ ocular tissues were observed around day 51; at this timepoint, 80.5% of the bimatoprost dosage was determined to have been released [72]. Over 99% of the drug dosage had been released by day 80 and tissue drug concentrations had declined significantly [72]. No studies in humans have been conducted as of yet testing the implant’s pharmacokinetics, pharmacodynamics, and other effects on the eye due to its novelty. The clinical studies are limited to the trials conducted for FDA approval.

Phase I/II trials followed 75 adult patients with POAG who received Bimatoprost SR dosed with 6, 10, 15, or 20 µg intracamerally following baseline washout in the study eye while the fellow eye received once-daily topical bimatoprost 0.03% [67,68]. The mean IOP reduction from baseline at 16 weeks was 7.2, 7.4, 8.1, and 9.5 mmHg for the 6-μg, 10-μg, 15-μg, and 20-μg doses, respectively, compared to 8.4 mmHg in the fellow control eyes [68]. Additionally, through week 16, over 90% of the eyes that received an implant did not require rescue treatment [68]. At 24 months, the mean IOP reduction from baseline was 7.5, 7.3, 7.3, and 8.9 mmHg for the 6-μg, 10-μg, 15-μg, and 20-μg doses, respectively, compared to 8.2 mmHg in the fellow control eyes [67]. A single administration of the implant was shown to control IOP in the majority of study patients for up to six months without retreatment [68]. Incidence of adverse events was similar between the implant eyes and the fellow control eyes two days after the implantation procedure, indicating the association of these events with the procedure rather than the implant [67,68]. Additionally, the majority of implants had either fully biodegraded or were less than a quarter of their initial size by 12 months [67]. For all doses, 82.9% of patients reported they were very or extremely likely to have another implant procedure if given the choice, and 88.6% reported they would recommend the procedure to others with glaucoma [67].

Phase III trials followed 594 adult patients with POAG who received either Bimatoprost SR dosed with 10 or 15 µg in one eye or twice-daily topical timolol maleate 0.5%, with 198 patients in each group [69]. The implant was readministered after 16 and 32 weeks; eyes administered with topical timolol received sham implantation procedures at the same timepoints of implantation. Both dose strengths of Bimatoprost SR were found to be noninferior to timolol in IOP-lowering capacity after each administration [69]. The mean IOP and mean change in IOP from baseline in the implant group eyes was also found to be consistently lower than in the topical timolol group eyes [69]. Additionally, Kaplan-Meier analysis was used to estimate the probability of patients not requiring additional treatment for one year after the last and third administration of the implant. The study estimated 75.5% in the 10-µg dose group and 73.0% in the 15-µg dose group would not require additional treatment for one year after the last administration of the implant; the probability after the third administration that patients would not require additional treatment for one year was estimated to be 82.1% in the 10-µg dose group and 87.8% in the 15-µg dose group [69]. In comparison, the patients treated with topical timolol had an estimated probability of not requiring additional treatment for one year after the last sham administration of 88.9% and after the third sham administration of 95.2% [69]. In terms of safety, the implant eyes once again demonstrated a higher incidence of adverse events than the topical timolol eyes, but these seemed to be transient, occurring within two days of administration [69]. Additionally, implant eyes showed a greater loss of mean corneal endothelial cell density in comparison timolol-treated eyes, however, this loss was not significant [69]. Overall, risk-benefit analysis favoured the Bimatoprost SR 10-µg dose over the 15-µg dose [69].

Further study is planned or currently ongoing in patients with POAG or OHT to compare Bimatoprost SR with selective laser trabeculoplasty and with multidrug therapy regimens, as well as to further evaluate the implant’s safety and efficacy [66]. The next step in the development of Bimatoprost SR will be to obtain approval for multiple administrations to become a clinically relevant treatment option. Future studies will be necessary to better determine the role of Bimatoprost SR in the treatment of glaucoma.

## Sustained release therapies under clinical investigation

### Bimatoprost ocular ring

While Bimatoprost SR is the only approved SR drug delivery system, there are many others currently under investigation. One of these therapies is the Bimatoprost Ocular Ring which was also produced by Allergan. Unlike Bimatoprost SR, this device is inserted in the upper and lower fornices and consists of an inner polypropylene support structure with an outer silicone matrix containing 13 mg of bimatoprost [65,73]. This extraocular insert has a targeted duration of six months and has currently completed Phase II of testing [65]. Overall, the Phase II trial indicated that the bimatoprost ring was non-inferior to topical timolol at two of the nine study timepoints, however, analysis deemed the study underpowered for the observed treatment effect [65,73]. The study enlisted 130 patients who were randomized to either the bimatoprost ring plus artificial tears twice-daily or a placebo insert plus topical timolol 0.5% twice-daily [73]. Patients in both groups were found to display clinically relevant reductions in IOP sustained across the 6-month study period, however, the patients receiving timolol saw an enhanced reduction in IOP as compared to patients receiving the bimatoprost ring [73]. Additionally, the bimatoprost ring was found to have met the study definition of non-inferiority to timolol at only two of the nine timepoints; non-inferiority was defined as when the upper bound of the 95% confidence interval exceeded the 1.5 mmHg limit [73]. In terms of safety and tolerance, the ring was deemed to be both safe and well tolerated. The retention ring of the ocular insert was 93.1% at 12 weeks and 88.5% at six months for both the bimatoprost ring and the placebo insert [73]. A following open-label extension study continuing off the Phase II trial found even higher retention rates of 97.3% and 94.7% for both of its cycle, suggesting that retention of the device increases with patient experience [74]. Additionally, the bimatoprost ring group did have a higher percentage of both ocular and non-ocular adverse events as compared to the timolol group, but these events were considered to be relatively mild or moderate in severity and transient [73]. The bimatoprost ring was also shown to display lower rates of conjunctival hyperaemia as compared to topical timolol solution [73]. The most commonly reported adverse events following the Phase II trial and the extension study were punctate keratitis in 16.0% of patients, eye discharge in 14.8%, and ocular discomfort in 6.2% [75]. The extension arm of the study also found that over 80% of participants found the ring to be comfortable and over 97% of participants found it to be tolerable [74,75]. The data from both of these studies showed that the bimatoprost ring could achieve sustained clinically relevant reduction in IOP over 19 months at most when applied at 6-month intervals and was both safe and tolerable with data indicating a preference for the bimatoprost ring over eye drops [74,75].

### iDose^®^

The intraocular implant iDose^®^ produced by Glaukos Corporation (San Clemente, California) is currently the furthest along in development with Phase III trials ongoing and set for completion and FDA approval in 2023 [76]. This titanium implant is placed intraocularly in the trabecular meshwork with a scleral anchor [65]. The implant releases a proprietary formulation of travoprost and has a targeted duration of six to 12 months [65,77]. The iDose implant requires surgical insertion and removal in the operating room [65]. It consists of three main parts which include a scleral anchor that is inserted into the inner wall of the sclera through the trabecular meshwork, the titanium body that serves as a reservoir for the drug, and a membrane that elutes the drug intracamerally [65]. The Phase II trial compared iDose at two different rates of elution to twice-daily topical timolol 0.5% [77]. The study enlisted 154 patients with 51, 54, and 49 randomized to the groups iDose TR fast-release arm, iDose TR slow-release arm, and timolol active comparator arm, respectively [76,77]. The study found the mean IOP reduction from baselines over the first 24 months was 7.9 mmHg or 29%, 7.4 mmHg or 28%, and 7.8 mmHg or 30% for the fast-release iDose group, slow-release iDose group, and timolol control group, respectively [76]. In addition to the device’s efficacy, the study indicated a very favourable safety profile with no clinically significant corneal endothelial cell loss, no corneal adverse events, and no adverse events of conjunctival hyperaemia in either of the iDose groups [76,77]. Currently, Glaukos Corporation is progressing towards enrolment completion for its ongoing Phase III clinical program; this study will consist of two prospective, randomized clinical trials designed to compare the safety and efficacy of the iDose device to topical timolol in reducing IOP in POAG or OHT patients with the primary endpoint of non-inferiority [76]. These trials are expected to include approximately 1,100 subject from over 100 clinical sites [76].

### ENV515

Another intraocular implant known as ENV515 developed by Envisia Therapeutics (Research Triangle Park, North Carolina) has completed Phase II testing. This rod-shaped device is placed intraocularly in the iridocorneal angle of the anterior chamber [64,65,78]. This implant consists of a biodegradable drug delivery system that releases travoprost and has a targeted duration of six to 12 months [65]. ENV515 is intended to be inserted in clinic but does not need to be removed [65]. No articles could be found describing the findings of the Phase II study, however, the company has made the trial’s data available on ClinicalTrials.gov. The study found a single dose of ENV515 decreased IOP by 6.7 ± 3.7 mmHg over 11 months [64,78]. Patients treated with ENV515 showed similar mean IOP reductions as compared to patients treated with topical travoprost 0.004% and patients treated with topical timolol 0.5% [65]. The most common adverse event was conjunctival hyperaemia which was associated with the implantation procedure. Preclinical studies in hypertensive and normotensive Beagle dogs also indicated ENV515 to have sustained IOP-lowering effects for 8 months after a single implantation and both favourable safety and tolerability [79,80].

### OTX-TP

Ocular Therapeutix (OTX) (Bedford, Massachusetts) designed an intracanalicular punctal plug, OTX-TP, for the delivery of travoprost to the ocular surface. Traditionally, punctal plugs, also known as lacrimal plugs, are commonly used to treat dry eye. By preventing the drainage of tears through the nasolacrimal duct, punctal plugs help maintain the volume of tears on the eye surface [81]. Punctal plugs have the benefit of being easily inserted and removed in a clinical setting while also being largely well-tolerated by patients. Recently, it has been suggested that punctal plugs could be utilized as SR drug delivery systems by loading the plug with a specific drug, allowing the eluted drug to mix with the tear fluid for delivery to the eye surface and intraocular tissues with greater efficacy and bioavailability than eye drops.

Based on a polyethylene glycol (PEG) hydrogel with brimonidine polylactic acid (PLA), the OTX-TP was designed to capitalize on the benefits of punctal plug delivery systems [64]. Having the benefit of outpatient placement and exchange, primary feasibility studies found OTX-TP was well-tolerated with a 100% retention rate over 10 days and a sustained IOP-lowering effect over a one-month period [82]. More recently, a prospective, multicenter Phase III clinical trial failed to achieve a primary endpoint of statistically significant IOP reduction from baseline compared to placebo at all nine study timepoints, finding statistically significant reduction in only eight of the nine time points, ranging from 3.27 to 5.72 mmHg reduction from baseline with greater reduction at earlier time points [83]. This suggests that this technology may hold promise in lowering IOP in POAG and OHT patients. Additionally, in this study of 554 subjects with POAG or OHT, the OTX-TP punctal plug was still well-tolerated, with episodes of canaliculitis and lacrimal structure disorder similar to rates in the placebo group [83].

### Evolute

Mati Therapeutics (Austin, Texas) is also developing a punctal plug delivery system known as Evolute for POAG as well as for allergy relief and nonsteroidal anti-inflammatory drug delivery. When loaded with latanoprost for POAG patients, two recently completed Phase II trials demonstrated retention rates of over 90% at 12 weeks and pressure reduction of 7 mmHg compared to 5 mmHg in topical latanoprost controls [64,84]. Previously, Mati Therapeutics acquired QLT’s Latanoprost Punctal Plug Delivery System, which has been shown to have statistically significant mean IOP reduction of 5.7 mmHg over four weeks in a clinical trial [85].

### Other SR therapies

Several other SR therapies are now beginning the clinical trial process for eventual approval. OTX began Phase I clinical trials for a bioresorbable travoprost intracameral implant known as OTX-TIC which is implanted in the anterior chamber to study device efficacy, durability, safety, and tolerability in POAG and OHT patients. This device has a targeted duration of four to six months [65]. Additionally, PolyActiva’s (Parkville, Australia) latanoprost free acid SR biodegradable implant is currently undergoing a Phase I clinical trial. This device is implanted in the iridocorneal angle with a targeted duration of 6 months [65].

These devices each demonstrate significant benefits for patients with POAG and OHT in reducing IOP without the challenges posed by topical therapies. However, further clinical research will be needed to better understand each of these systems once approved before they become a mainstay in the treatment of glaucoma. In addition to these devices, there are many emerging technologies that may represent the future of glaucoma management and SR therapy.

## Emerging technologies

### Contact lens drug delivery systems

There is a depth of emerging research that is aimed at developing alternative SR therapies for glaucoma, including contact lens delivery systems. As opposed to topical eye drops, which generally have a bioavailability less than 5%, contact lenses present a favourable alternative as they are able to be placed directly on the cornea separated only by the post-lens tear film [81]. Contact lenses purport to present several advantages to topical eye drops. Contact lenses may retain drugs in the tear film for upward of 30 min, versus two minutes for topical eye drops, a time difference which may increase drug bioavailability by upwards of 50% [86–88]. Additionally, contact lenses present advantages related to ease of wear, direct contact with the ocular surface, and hydrogel composition. Traditional hydrogel contact lenses allow for molecular movement of water and nutrients to the corneal surface, so approaches using this delivery system have focused on soaking hydrogel lenses in concentrated solutions of active pharmaceuticals [81].

Published data on contact lens drug delivery systems are largely from animal models which generally explored the feasibility of using lenses for extended periods [81]. One such trial studied latanoprost-releasing SR contact lenses in a glaucomatous monkey model. In this trial, two different lenses, one low-dose and one high-dose, were prepared by lathing a latanoprost-polymer film into the contact lens hydrogel, and then tested against a control of topical daily latanoprost [89]. The researchers found that one-week usage of both high and low-dose contact lenses had diurnal IOP drops of 6.0 to 10.2 mmHg and 4.0 to 7.8 mmHg, respectively, compared to 2.9 to 6.6 mmHg for the controls, leading to the conclusion that SR latanoprost delivery by contact lenses showed similar efficacy to daily latanoprost application [89]. Similarly, silicone contact lenses containing timolol have shown the ability to release timolol over a one-month period in Beagle dogs with a favourable IOP-lowering effect [90]. Importantly, uptake studies of silicone contact lenses have shown different methods of drug loading have different efficacies as well as different burst release profiles. According to Yan et al. [91], molecular imprinting of contact lenses with bimatoprost showed better efficacy than conventional soaking methods, as well as better burst release profiles and SR release rate profiles in an *in vivo* rabbit tear fluid model [91].

Other SR contact lens technologies are still in early stages of development including micelles-loaded contact lenses, chitosan-based nano-coatings, diamond nano-gel embedded lenses, lenses with embedded microtubes as drug containers, and temperature sensitive lenses [92–97]. Additionally, polymeric films similar to contact lenses are being developed as delivery platforms for glaucoma medications [98]. Researchers have tested hyaluronic acid, a natural component of eye fluid with adhesive properties, formulations combined with hydroxypropyl methylcellulose, a thickening agent commonly used in eye drops, to form erodible ocular films that can be drug-loaded for SR delivery of IOP-lowering agents [98]. Similarly, a nanofiber patch containing timolol was able to sustain timolol delivery over a period of 72 h in albino New Zealand rabbits [99].

### Nanotechnology for SR drug delivery

Nanotechnology describes materials and devices that are scaled in the order of less than 100 nm. The objective medical application of nanotechnology is to design materials and devices that may augment human biology at a molecular level [100]. Recently, nanotechnology has been suggested as a potential revolutionary force in the approach to challenges in ophthalmology, including in the design of SR drug delivery systems for glaucoma.

One such application is polymeric hydrogels, which are three-dimensionally organized molecular frameworks that enable drug molecules to be diffused throughout the entire structure, outfitting the hydrogel as a drug reservoir [101]. In addition to being transparent and highly biocompatible given their flexibility and high water content, polymeric hydrogels are generally well-adherent to the ocular surface leading to increased contact time and patient compliance by overcoming challenges associated with high dose frequency [102,103]. As biocompatible nanotechnology, polymeric vehicles can reduce both dosing regimen or adequate concentration by overcoming the lipophilic barrier of the corneal epithelium [104].

When augmented with bioadhesive agents, polymeric hydrogels have increased ocular residual time and bioavailability. Lei et al. [105] investigated topical delivery of brimonidine and levofloxacin using covalently cross-linked chitosan hydrogel sheets for SR drug delivery up to a period of 24 h [105,106]. In 2015, Malavia et al. [107] developed a dissolvable hydrogel template and loaded OHR1031, a small drug molecule. They found the hydrogel template allowed for near 100% incorporation efficiency along with a SR delivery pattern close to zero order for more than three months with limited initial burst [107]. Similarly, Ilka et al. [108] designed nanogel biopolymers for the release of timolol maleate which showed burst release in the first hour but then a slower SR pattern over the following 24 h. More recently, Cuggino et al. [109] derived a nanogel that effectively loaded timolol, delivered the drug in a sustained pattern, and successfully lowered IOP in a rabbit glaucoma model over a period of 48 h.

One example of a polymeric hydrogel is a microsphere [110]. As early as 2009, Bertram et al. [111] detailed PLGA/poly(lactic acid) (PLA) microspheres (MS) loaded with a formulation of timolol maleate that was able to deliver the drug continuously over a period of 107 days. In 2017, Fedorchak et al. [112], investigated loading PLGA MS with brimonidine tartrate and incorporating them into hydrogels for SR treatment of glaucoma. Developed by Otero Therapeutics, this technology is called SoilDrop and is currently undergoing preclinical trials. Most recently, they determined a single drop of the non-invasive polymeric hydrogel maintained *in vivo* efficacy over 28 days as compared to twice-daily topical brimonidine drops in a rabbit glaucoma model [112]. Other studies with drug-loaded MS have shown their potential use in SR drug delivery both *in vitro* and *in vivo* [113–116].

Another form of polymeric hydrogels is dendrimer nanofiber. Dendrimers, also known as arborols, cascade molecules, and starburst polymers, are nanoscale polymers composed of multivalent molecules with branched structures. They are proposed for SR drug delivery as their structures contain three different sections that can be functionalized for drug delivery [81]. The most common form under investigation for SR drug delivery are polyamidoamine (PAMAM) dendrimers, which are historically cytotoxic but can be modified to combat this. In a 2017 study, Lancina et al. [117] prepared a PAMAM dendrimer with brimonidine tartrate to form a fast-dissolving nanofiber mat. Over a three-week period, they found IOP response was similar between the nanofiber mat and daily topical brimonidine tartrate in a Brown Norway rat model, noting immediate dissolution upon placement, favourable biocompatibility, and efficacious drug delivery [117]. Ultimately, polymeric ophthalmic hydrogels have been found to be well-tolerated and biocompatible in animal models and human trials [117–121].

Aside from hydrogels, there are several other applications of nanotechnology that are being evaluated for glaucoma therapy. Microemulsions, which are clear and stable formulations of water and oil, are one possible application given their ability to improve corneal contact time and easily incorporate and deliver drug molecules in a SR pattern, though they are limited by biocompatibility issues [122,123]. Nanosuspensions, which are formulations of drugs with poor water solubility stabilized in a medium, have elevated bioavailability [103]. Additionally, nanoemulsions, which are small compositions of oil, water, and surfactant, are able to cross cell membranes with minimal toxicity and adverse effects. Catioprost, a latanoprost-loaded nanoemulsion vehicle called navasorb, is currently undergoing Phase III clinical trials [103].

In addition to suspensions and emulsions, other nano-pharmaceuticals are in development for the management of glaucoma. Nanoparticles are promising in their ability to overcome ocular structural barriers while maintaining structural integrity [103]. Studies have demonstrated nanoparticles loaded with drug formulations may increase bioavailability with efficient IOP reduction [124–128]. Recently, Barwal et al. [129] formulated nanoencapsulated brimonidine to create ultra-small nanoparticles that would improve drug efficacy and reduce side effects. When added to trabeculectomy tissue of glaucoma patients, they identified better dilation of the meshwork, indicating increased bioavailability of the drug through this modality [129]. Of nanoparticles under investigation, lipid nanoparticles demonstrate enhanced biocompatibility, drug permeability, and IOP reduction [103,130–132]. Lipid nanoparticles loaded with brimonidine have been shown to increase affinity towards the cornea, SR drug delivery, and significant IOP reduction without toxicity as compared to topical brimonidine [133].

Nanotechnology is also being used to explore innovative vesicular delivery systems. Vesicles present the ability to control drug delivery by avoiding enzymes at the tear film or corneal epithelial surface. So-called nanovesicles are able to deliver hydrophilic and hydrophobic drugs in a stabilized manner with high efficacy and reduced drug toxicity [103]. So far, latanoprost- and brinzolamide-loaded nanoliposomes have been evaluated, with formulations of timolol in gelatinized core liposomes under development [134–138]. A liposome formulation incorporating citicoline was recently shown to carry the compound to the ONH and retina [139].

Aside from liposomes, there are many other forms of nanovesicles under development [103]. Among these, cubosomes, which are nanostructures made from the cubic liquid crystalline phase of lipids, may be most adaptable for SR drug delivery due to increased stability compared to liposomes and the ability to load and deliver a higher capacity of drugs [103]. Studies have demonstrated cubosomes loaded with various drugs to have greater corneal permeability and prolonged IOP-lowering capacity with an SR delivery profile [140,141]. Additionally, leciplex, which are cationic phospholipid-based, and niosome, which are non-ionic surfactant-based, nanovesicle formulations have demonstrated SR efficacy in glaucomatous rabbit models [142–144].

In addition to vesicular systems and hydrogels, there are several novel nanotechnology-based drug delivery systems that may be promising, including *in situ* assembly systems, emulsomes, and gold nanoparticles [103]. Schnichels et al. [145] developed an *in situ* assembly system leveraging DNA nanotechnology, in which micelle nanoparticles form themselves out of DNA molecules modified with lipid moieties. Previously, these kanamycin-loaded nanoparticles were used to treat acute corneal infection while optimising ocular adhesion [146]. When this same technology was loaded with travoprost, increased ocular residence time and improved biocompatibility were noted [145].

Gold nanoparticles may provide an ultra-stable platform for antiglaucoma medication delivery without cytotoxicity [147]. Additionally, gold nanoparticles loaded with timolol may be combined with contact lenses to deliver drugs in an SR pattern, overcoming notable issues associated with contact lens drug delivery systems including low drug loading, burst release patterns, and maintenance of critical contact lens functions and properties [148].

While advances in nanotechnology are encouraging, there are still considerable challenges to overcome including cytotoxicity concerns, pharmacokinetic and dynamic questions, and development of stabilisation and sterilisation techniques. [103,149,150]. Further research and development are necessary, however, nanotechnology is a promising avenue for the future of glaucoma therapies.

## Challenges for SR technology

As SR drug delivery systems are developed and tested, a variety of questions and challenges must be answered before they can become a mainstay in glaucoma treatment. Chief among these are questions surrounding drug efficacy, safety profiles, and differences in delivery routes and how these considerations compare to conventional topical therapies.

In general, the SR therapies currently under clinical investigation tend to display higher incidences of adverse events, but their safety profiles are favourable. The most common concern for these devices seems to be conjunctival hyperaemia associated with the implantation procedure. However, this issue has been repeatedly shown to be transient for multiple devices, not dissimilar to topical hypotensive therapies. These are commonly known to have a relatively high incidence of both ocular and non-ocular adverse events of minor severity . Additionally, these devices have been found to be both comfortable and tolerable for patients. Long-term studies are needed, however, to appropriately gauge patient comfort for extensive treatment regimens. However, one issue with more lengthy clinical trials is the emergence of SR toxicity concerns. This is a major challenge for all SR technologies, which is further complicated by the individualisation of these issues to each device. Cytotoxicity is a major concern that must be addressed to properly evaluate the long-term safety and efficacy of these devices in establishing them as primary clinical options for glaucoma management. It must also be noted that more lengthy trials for these SR devices may also require much higher development costs as compared to traditional therapies.

For each drug delivery system, pharmacokinetic and bioavailability questions are at the forefront of determining the role of SR drug delivery systems in glaucoma therapy. Current studies modelling drug delivery require a variety of rate constants that are difficult to estimate without experimental data [81]. Additionally, pharmacokinetics and bioavailability may also be dramatically impacted by the different drug delivery modalities.

Finally, these SR drug delivery devices currently under clinical investigation must also be compared with topical prostaglandin analogues to resolve remaining questions about their efficacy. Currently investigated SR devices all deliver a prostaglandin analogue, but non-inferiority comparisons to timolol are not ideal in understanding the role of these devices in glaucoma management. Timolol is the historical comparator for FDA approval, so further clinical studies are needed to better understand how these novel devices compare to topical prostaglandin analogues, combined or multidrug therapies, and surgical and laser alternatives in terms of long-term efficacy and safety.

As the development of SR drug delivery systems continues, the unique challenges inherent to glaucoma must also be considered. Glaucoma is a complex, multifactorial disease in which IOP is only one risk factor for onset and progression; other risk factors, including demographic characteristics, co-morbidities, and ocular structural and vascular predispositions, must also be considered to effectively treat the disease [1,151]. Additionally, glaucoma is a dynamic disease with IOP known to fluctuate diurnally, which may present an additional factor to consider in disease management [152,153]. Finally, current glaucoma management generally consists of targeted IOP reduction through initial topical monotherapies. Given the full armament of glaucoma therapies in the hands of clinicians, it is unclear how SR drug delivery systems will interplay with topical therapies to achieve individualised disease management.

## Conclusion

With the limitations in adherence and efficiency associated with topical drug delivery, the future of glaucoma management points towards the development and utilisation of SR drug delivery devices. The potential for these devices to improve patient adherence may dramatically impact disease progression and patient quality of life. While only one drug delivery device has obtained FDA approval for the treatment of glaucoma, there are several others undergoing clinical trials and many in pre-clinical stages with potential to shape future glaucoma therapy. Nanotechnology and other increasingly specialized technologies currently in preclinical testing have the potential to revolutionize the management, and prognosis, of glaucoma.

While many questions remain, the widespread use of SR therapies may be a reality within the next five years, and these therapies could eclipse traditional topical eye drop delivery by the end of this decade [1]. Importantly, however, the literature has begun shifting from a predominately IOP-only perspective to one embracing the multifactorial nature of glaucomatous progression. Further developments in glaucoma therapies and specifically SR drug delivery will need to consider these ramifications to facilitate truly individualized medication regimens. As healthcare systems adopt a value-based personalized medicine emphasis, clinicians should be prepared to consider these treatment modalities not just for non-adherent patients but for all patients to most effectively adress the multifactorial nature of glaucoma. 

## Data Availability

Data sharing is not applicable to this article as no new data were created or analysed in this study.
